# Sun protection patterns among organ transplant recipients and nonorgan transplant patients with skin cancers in Singapore

**DOI:** 10.1016/j.jdin.2024.05.001

**Published:** 2024-06-14

**Authors:** Li Houng Chen, Shashendra Aponso, Choon Chiat Oh

**Affiliations:** a Resident, Internal Medicine, Singapore Health Services, Singapore; b Department of Dermatology, Singapore General Hospital, Singapore; c Duke-NUS Medical School, Singapore

**Keywords:** organ transplant, Singapore, skin cancer, Southeast Asia, sun protection

*To the Editor:* Organ transplant recipients (OTRs) are at higher risk of developing cutaneous malignancies, especially squamous cell carcinoma.^1^ As sun exposure is a risk factor for squamous cell carcinoma, sun protection is crucial in OTRs. Sun protection has neither been reported in transplant patients in Singapore, nor their dermatologic quality of life after skin cancer events. Present studies largely studied sun protection in OTRs with fairer skin type/Caucasians. Our study offers insight into sun protection practices of Asian and darker-skinned patients.

We studied 63 adults (transplant and nonorgan transplant recipients) with cutaneous squamous cell carcinoma who were seen at the dermatology clinic in a tertiary hospital from January 2019 to March 2022 (Table I). They were surveyed regarding sun protection practices, history of full-body skin checks and completed the dermatology life quality index questionnaire. Univariate analysis with chi-square test was performed to compare categorical data between patient groups and examine associations between transplant status and sun protection behaviour.

**Table I:** Baseline demographics of the 2 groups

	Frequency	
	Transplant (*n* = 24)	Control ie, nontransplant (*n* = 39)
Gender		
Female	14 (58.3%)	10 (25.6%)
Male	10 (41.7%)	29 (74.4%)
Ethnicity		
Chinese	24 (100%)	35 (89.7%)
Malay	0	1 (2.6%)
Indian	0	1 (2.6%)
Others	0	2 (5.1%)
Age		
Below 65	6 (25%)	3 (7.7%)
65 and above	18 (75%)	36 (92.3%)
Skin colour type		
1	0	2 (5.1%)
2	5 (20.8%)	13 (33.3%)
3	7 (29.2%)	7 (17.9%)
4	12 (50%)	17 (43.6%)

38.1% (*n* = 24) of respondents were OTRs and 61.9% (*n* = 39) were controls. 68.3% (*n* = 43) had skin phototype 3 or darker. Overall, there was poor utilization of sun protection in both groups (Table II). Sunscreen uptake was particularly poor with 74.4% (*n* = 29) of patients in the control group and 75% (*n* = 18) of those in the transplant group “never” wearing sunscreen. Asians and darker-skinned individuals have lower perceived risk of skin cancer and poorer usage of sun protection.^2^ Singaporeans too have low national awareness of the importance of sun protection.^3^ Characteristics of sunscreen can also be prohibitive— the appearance of a white cast which is more obvious on darker and olive skin tones, and its greasy texture.^4^ The cost of sunscreens might also be a deterrent. Asians with darker skin types are less likely to sunburn,^4^ which might perpetuate the belief that sunscreen is unnecessary.

**Table II:** Results between demographic variables and sun protection behavior

	DLQI (Mean)	Sunscreen usage			Umbrella usage			Sunglass usage			Hat usage			T shirt usage			Suntan habit			Sunburn		Self-skin check			Professional check		
		Yes	No	*P*-value	Yes	No	*P* value	Yes	No	*P* value	Yes	No	*P* value	Yes	No	*P* value	Yes	No	*P* value	Yes	No	Yes	No	*P* value	Yes	No	*P* value
Gender																											
Female	2.7	10	14	.0199	14	10	.124	8	16	1.0	5	19	.835	22	2	.582	4	20	.672	0	24	6	18	.677	13	11	.155
Male	2	6	33		15	24		13	26		9	30		34	5		5	34		1	38	8	31		14	25	
Ethnicity																											
Chinese	2.3	15	44	.437	29	30	-	20	39	.637	12	47	-	52	7	-	8	51	.153	1	58	14	45	-	26	33	.868
Non-Chinese	2	1	1		0	2		1	1		2	0		4	0		1	1		0	2	0	2		1	1	
Age																											
<65 y	2.8	1	8	.288	3	6	.409	2	7	.445	2	7	1.00	8	1	1	2	7	.462	0	9	5	4	.00938	6	3	.119
≥65 y	2.2	15	39		26	28		19	35		12	42		48	6		7	47		1	53	9	45		21	33	
Skin colour type																											
1	0.5	1	1	.507	2	0	-	2	0	-	0	2	-	2	0	-	0	2	-	0	2	0	2	-	0	2	-
2	1.6	6	12		11	7		6	12		5	13		17	1		5	13		1	17	2	16		7	11	
3	2.7	4	10		5	9		6	8		2	12		12	2		0	14		0	14	3	11		8	6	
4	2.7	5	24		11	18		7	22		7	22		26	3		4	25		0	29	9	20		12	17	
Transplant status																											
Transplant	2.4	6	18	.955	11	13	.980	7	17	.582	5	19	.835	22	2	.582	3	21	.751	1	23	9	15	.0221	14	10	.0515
Control	2.2	10	29		18	21		14	25		9	30		34	5		6	33		0	39	5	34		13	26	

Positive response: (1) for sun protection practices, positive response defined as “rarely” to “always”, (2) self/partner skin check defined as patient or partner examining their entire body including their back the last 12 months from time of survey, (3) professional skin checks positive response defined as skin checked for skin cancer from head to toe by a health professional. Significance level taken at 0.05.

There was significant association between self-skin check and history of organ transplant (*P* = .0221), possibly due to increased awareness of cutaneous malignancies. Studies have shown the effectiveness of a dedicated dermatology transplant clinic in improving sun protection.^5^ All solid organ transplant patients in our institution are referred to the dermatology service and seen at least annually for a full-body skin check. Physician-led counselling on skin health is performed extensively during their first visit and reinforced during follow-up visits. There was no other statistically significant difference between sun protection patterns in OTRs and nontransplant patients (Table II).

OTRs face a multitude of problems in the peri-transplant period which renders sun protection an unlikely priority. Appropriate timing of sun protection education is crucial to prevent information overload during a time of heightened emotions.

Mean dermatology life quality index scores were low at 2.23 and 2.42 for the control and transplant groups respectively, possibly due to the localized nature of the skin tumours.

In conclusion, systemic measures such as reducing the cost of sunscreen with reimbursements should be considered to improve sunscreen usage. Dedicated transplant dermatology clinics are effective in improving sun protection behaviour. Physicians should deliver empathetic care and individualized education while paying attention to transplant patients’ ever-changing healthcare needs.

## Conflicts of interest

None disclosed.
